# Prevalence of carbapenemase-producing Enterobacteriaceae from human clinical samples in Ethiopia: a systematic review and meta-analysis

**DOI:** 10.1186/s12879-023-08237-5

**Published:** 2023-05-03

**Authors:** Ermiyas Alemayehu, Temesgen Fiseha, Alemu Gedefie, Nuhamin Alemayehu Tesfaye, Hussen Ebrahim, Endris Ebrahim, Mesfin Fiseha, Habtye Bisetegn, Ousman Mohammed, Mihret Tilahun, Daniel Gebretsadik, Habtu Debash, Mengistie Yirsaw Gobezie

**Affiliations:** 1 Department of Medical Laboratory Sciences, College of Medicine and Health Sciences, Wollo University, Dessie, Ethiopia; 2 Department of Pharmacy, College of Medicine and Health Sciences, Wollo University, Dessie, Ethiopia

**Keywords:** Prevalence, Carbapenemase, Enterobacteriaceae, Ethiopia, Systematic review, Meta-analysis

## Abstract

**Introduction:**

Carbapenemase-producing Enterobacteriaceae are by far the most public health and urgent clinical problems with antibiotic resistance. They cause longer hospital stays, more expensive medical care, and greater mortality rates. This systematic review and meta-analysis aimed to indicate the prevalence of carbapenemase-producing Enterobacteriaceae in Ethiopia.

**Methods:**

This systematic review and meta-analysis was conducted based on Preferred Reporting Items for Systematic Reviews and Meta-Analysis guidelines. Electronic databases like PubMed, Google Scholar, CINAHL, Wiley Online Library, African Journal Online, Science Direct, Embase, ResearchGate, Scopus, and the Web of Sciences were used to find relevant articles. In addition, the Joanna Briggs Institute quality appraisal tool was used to assess the quality of the included studies. Stata 14.0 was used for statistical analysis. Heterogeneity was assessed by using Cochran’s Q test and I^2^ statistics. In addition, publication bias was assessed using a funnel plot and Egger’s test. A random effect model was used to estimate the pooled prevalence. Sub-group and sensitivity analysis were also done.

**Results:**

The overall pooled prevalence of carbapenemase-producing Enterobacteriaceae in Ethiopia was 5.44% (95% CI 3.97, 6.92). The prevalence was highest [6.45% (95% CI 3.88, 9.02)] in Central Ethiopia, and lowest [(1.65% (95% CI 0.66, 2.65)] in the Southern Nations and Nationalities People Region. In terms of publication year, 2017–2018 had the highest pooled prevalence [17.44 (95% CI 8.56, 26.32)] and 2015–2016 had the lowest [2.24% (95% CI 0.87, 3.60)].

**Conclusion:**

This systematic review and meta-analysis showed a high prevalence of carbapenemase-producing Enterobacteriaceae. So, to alter the routine use of antibiotics, regular drug susceptibility testing, strengthening the infection prevention approach, and additional national surveillance on the profile of carbapenem resistance and their determining genes among Enterobacteriaceae clinical isolates are required.

**Systematic review registration:**

PROSPERO (2022: CRD42022340181).

**Supplementary Information:**

The online version contains supplementary material available at 10.1186/s12879-023-08237-5.

## Introduction

Bacterial antimicrobial resistance (AMR) is one of the major public health problems in the 21st century. It happens when changes in bacteria make the medications used to treat infections less effective [1]. In 2019, there were an estimated 4.95 (95% uncertainty level [UI], 3.62–6.57) million deaths associated with bacterial AMR, of which 1.27 million (95% UI, 0.911–1.71) were attributable to bacterial AMR. *Escherichia coli*, and *Klebsiella pneumoniae* were among the six most common pathogens associated with resistance-related deaths [2].

Infections caused by multidrug-resistant Enterobacteriaceae, like extended-spectrum-lactamase producing Enterobacteriaceae have been successfully treated with carbapenem antibiotics for a long time [3]. Carbapenems contain a beta-lactam ring that makes them more stable against the majority of β-lactamases [4]. According to Clinical Laboratory Standards Institute (CLSI) guidelines, meropenem, imipenem, ertapenem, and doripenem are used as therapies for infections caused by Enterobacteriaceae [5]. The emergence of Enterobacteriaceae producing carbapenemases has resulted in widespread resistance to carbapenems [6]. The production of carbapenemase enzymes, which are encoded by numerous genes and can be transmitted between Enterobacteriaceae via transferable genetic elements, is the primary mechanism for the development of carbapenem resistance in Enterobacteriaceae. From these, Class A *Klebsiella pneumoniae* carbapenemase, Class B metallo-lactamase, and Class D OXA-lactamase are examples of commonly encountered enzymes [7].

The World Health Organization identifies Enterobacteriaceae as a significant category that causes drug-resistant illnesses [8, 9]. The Centers for Disease Control (CDC) also described that CRE, such as *Klebsiella* species, *Escherichia coli*, and *Enterobacter* species are the most important developing resistance threats worldwide [10].

Isolates of Enterobacteriaceae that produce carbapenemase frequently exhibit multi-resistant strains due to their resistance to a wide range of different beta-lactam and non-beta lactam antibiotics [11]. From the standpoint of public health, carbapenemase-producing isolates are by far the most urgent clinical problem with antibiotic resistance [3]. There has been an alarming increase of carbapenem-resistant Enterobacteriaceae in recent years, predominantly *K. pneumoniae* [12, 13]. Compared to carbapenem susceptible Enterobacteriaceae, carbapenem-resistant Enterobacteriaceae (CRE) infections cause longer hospital stays, more expensive medical care, and greater mortality rates [14]. Therefore, the increasing prevalence of carbapenemase-producing strains is a significant issue, particularly in nations like Ethiopia [15].

Carbapenems are currently being used more frequently in Ethiopian healthcare institutions or by doctors as an empirical treatment. Because of this, there are still few effective treatments for severe CRE infections [16]. Emergences of Enterobacteriaceae that are resistant to carbapenems are a significant medical issue. The majority of countries are at risk of becoming the next victims of CRE. In order to stop the spread of such resistant microbes, infection prevention and control systems should be reinforced [17]. Therefore, this systematic review and meta-analysis aimed to estimate the pooled prevalence of carbapenemase-producing Enterobacteriaceae in Ethiopia.

## Methods

### Reporting and protocol registration

This Systematic Review and meta-analysis was reported using Preferred Reporting Items for Systematic Reviews and Meta-Analyses (PRISMA) guidelines [18]. The protocol was registered at International Prospective Register of Systematic Reviews (PROSPERO) with registration number of CRD42022340181.

### Data sources and search strategies

Systematic searches of electronic databases such as PubMed, Google Scholar, CINAHL, Wiley Online Library, African Journal Online, Science Direct, Embase, ResearchGate, Scopus, and the Web of Sciences were used to retrieve potentially eligible studies reporting the prevalence of carbapenemase-producing Enterobacteriaceae (CPE) in Ethiopia. In addition, the proceedings of annual research conferences and university repositories were screened. A snowball search was also conducted using the bibliographies of the identified studies to include additional relevant studies omitted during electronic database searches. The search was conducted from May 30, 2022, to July 15, 2022.

The following combination of key words were used to access all potentially eligible studies: “prevalence” OR “epidemiology” AND “carbapenemase-producing isolates” OR “CPE” OR “CRE” OR “carbapenem resistant” OR “multidrug-resistant” OR “antimicrobial resistance” AND “Enterobacteriaceae” OR “gram-negative bacteria” AND “Ethiopia”. The Boolean operators’ terms “OR” and “AND” were used as necessary.

### Eligibility criteria

#### Inclusion criteria

Articles that fulfilled the following criteria were included in the final analysis: original articles published in peer-reviewed journals or grey literature, observational studies (cohort, cross-sectional and case control), articles published in English language, studies that reported prevalence of CPE in any region of Ethiopia, studies involving human/clinical samples, studies that accurately report the bacterial isolates of Enterobacteriaceae and their carbapenem resistance pattern based on the Clinical and Laboratory Standards Institute (CLSI), and European Committee on Antimicrobial Susceptibility Testing (EUCAST) guidelines, studies published until July 15, 2022 were included.

#### Exclusion criteria

Qualitative studies, review articles, case reports, narrative reviews, conference abstracts with no full information or if authors have not responded to our inquiry on the full text, editorials, commentaries, letters to the editor, author replies, studies not involving human/clinical samples and studies that do not include quantitative data on the prevalence of CPE were excluded.

### Study selection

EndNote version 20 software was used to import all articles that were found through searching electronic databases, conference proceedings, and the bibliographies of identified studies. Then, duplicates were eliminated. Based on the eligibility criteria, the title, abstract, and full text of each article were carefully screened by two independent reviewers. Disagreements between the two reviewers were settled through discussion of the inclusion of a third reviewer to select articles for the final review.

### Quality assessment

Independent reviewers critically analyzed the included studies to make sure the findings were reliable and consistent. The Joanna Briggs Institute (JBI) quality appraisal tool adapted for cross-sectional studies was used to assess the quality of the included studies [19]. The tool consisted of eight criteria. Studies with a quality score of 50% or higher were considered to be of good quality and were included for the analysis.

### Data extraction process

The required information were extracted and summarized using an extraction sheet in Microsoft Office Excel software. The key findings regarding the prevalence of CPE were extracted by three independent reviewers. The Microsoft Excel sheet was prepared under subheadings decided upon by all reviewers. The three reviewers cross-checked their findings carefully, and disagreement was resolved by discussion and repetition of the steps when necessary. The extracted data contains the name of the first author, publication year, region where the study was conducted, study area, study design, study population, sample size, diagnostic methods, specimen types, species of Enterobacteriaceae isolates, number of CPE isolates, and prevalence of CPE isolates.

### Statistical methods and analysis

The extracted data was exported to STATA version 14.0 for statistical analysis. Cochran’s Q test and I^2^ statistics were used to quantify and assess the presence of heterogeneity between studies. The presence of heterogeneity was defined as I^2^ test statistic values greater than 50% [20] and p-value results from a Q test less than 0.05. A random effect model was used to estimate the pooled prevalence of CPE with a 95% confidence interval [21]. The results were presented using a forest plot. The funnel plot and Egger weighted regression test were used to assess the presence or absence of publication bias. The asymmetry of the funnel plot and a p-value of < 0.05 in Egger’s test were suggestive of the presence of significant publication bias. In addition, subgroup analysis was carried out based on region, year of publication, and city. Furthermore, sensitivity analysis was also performed to determine the impact of a single study on the overall pooled estimate.

## Results

### Description of included studies

**Fig. 1:**
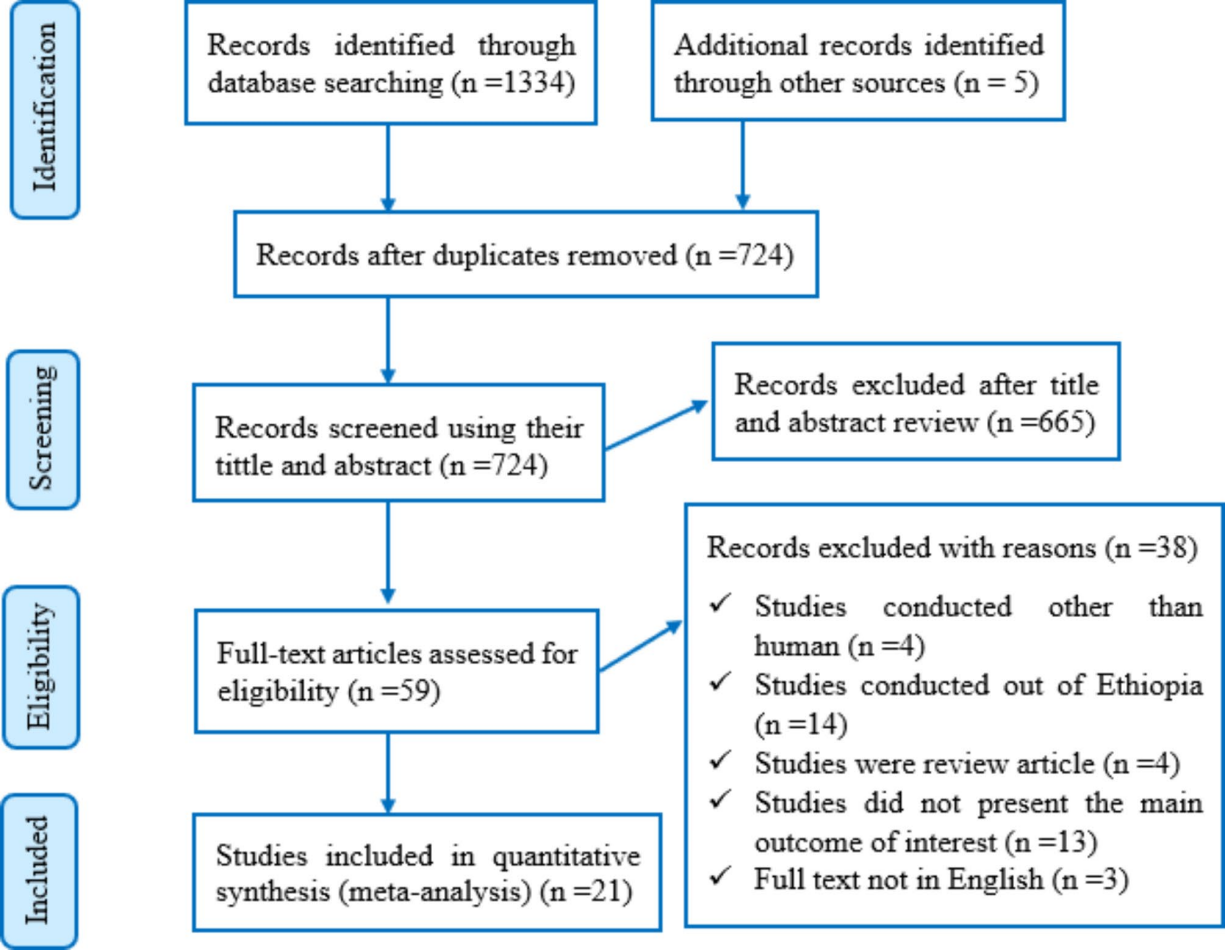
Flow diagram of the included studies for the systematic review and meta-analysis of the prevalence of CPE in Ethiopia

Database searches and other sources yielded a total of 1339 articles. From those articles, duplication resulted in the removal of 615 articles. A total of 724 papers were scrutinized for their titles and abstracts, and 665 studies were eliminated. A total of 59 full-text articles were then reviewed against the eligibility criteria. Following that, 38 full-text articles were excluded. Finally, only 21 articles were deemed potentially eligible and included in this review for the final analysis (Fig. 1).

### Characteristics of the included studies

A total of 21 original articles reporting studies conducted in different regions of Ethiopia were included in this systematic review and meta-analysis (Tables 1 and 2). All the studies had a quality score greater than 50%. The majority of the included studies were reported from Addis Ababa (47.6%) [16, 22–30], followed by the Amhara Region (33.3%) [31–37]. Sidama [38] and the Oromia region [39] were represented by a single study. In terms of the study design, all studies were cross-sectional studies. A variety of clinical specimens, including blood, urine, stool, and other body fluids, were used by the authors. A total of 3,932 Enterobacteriaceae bacterial isolates were included. The studies reported numbers of isolated Enterobacteriaceae from different clinical samples ranging from 33 in Addis Ababa [26] to 404 in Gondar [36]. The highest prevalence of CPE (30.5%) was reported from Addis Ababa in 2019 [27], while the lowest (1%) was reported from Gondar in 2022 [36]. 

**Table 1:** Distribution and characteristics of studies on CPE in Ethiopia

Authors	Study area	Region	Pub year	Study design	Participants
Desta et al. [25]	Addis Ababa	Central	2016	Cross -sectional	All hospitalized patients
Legese et al. [26]	Addis Ababa	Central	2017	Cross -sectional	Patients suspected of septicemia and UTIs
Beyene et al. [24]	Addis Ababa	Central	2019	Cross -sectional	Referred samples
Mitiku et al. [27]	Addis Ababa	Central	2019	Cross -sectional	Septicemia suspected under five children
Desalegn et al. [30]	Addis Ababa	Central	2019	Cross -sectional	Referred patients
Abdeta et al. [29]	Addis Ababa	Central	2021	Cross -sectional	Referred samples
Seman et al. [22]	Addis Ababa	Central	2021	Cross -sectional	Patients affected by urinary tract infection (UTI)
Tekele et al. [16]	Addis Ababa	Central	2021	Cross -sectional	All patients from both impatient and outpatient clinics
Seman et al. [23]	Addis Ababa	Central	2022	Cross -sectional	Adults and pediatric patients
Awoke et al. [28]	Addis Ababa	Central	2022	Cross -sectional	All patients from both impatient and outpatient clinics
Aklilu et al. [40]	Arba Minch	SNNPR	2020	Cross -sectional	Hospitalized patients with gastrointestinal colonization
Zakir et al. [41]	Arba Minch	SNNPR	2022	Cross -sectional	Neonates in intensive care units
Eshetie et al. [37]	Gondar	Amhara	2015	Cross -sectional	Symptomatic UTI suspected patients
Moges et al. [31]	Bahir Dar	Amhara	2019	Cross -sectional	Patients suspected for having bloodstream, UTI, wound and others infections
Alebel et al. [32]	Bahir Dar	Amhara	2021	Cross -sectional	Patients in intensive care units with symptoms for UTI, wound and others
Moges et al. [35]	Gondar, Dessie, and Debre Markos	Amhara	2021	Cross -sectional	Patients suspected of having bloodstream, UTI, wound and other infections
Worku et al. [36]	Gondar	Amhara	2022	Cross -sectional	Gastrointestinal tract complaint patients
Amare et al. [34]	Gondar	Amhara	2022	Cross -sectional	Asymptomatic food handlers working at the University of Gondar cafeteria
Tadesse et al. [33]	Bahir Dar	Amhara	2022	Cross -sectional	Patients symptomatic for bacterial infections
Alemayehu et al. [38]	Hawassa	Sidama	2021	Cross -sectional	All patients who visited the microbiology laboratory
Gashaw et al. [39]	Jimma	Oromia	2018	Cross -sectional	Patients had culture confirmed healthcare associated infections

**Table 2:** Clinical characteristics of included articles describing CPE in Ethiopia

Authors	Sample size	Diagnostic methods	No of isolates	Bacterial species	No (Prev)
Desta et al. [25]	267	ROSCO Neo-Sensitabs	267	*E. coli, K. pneumoniae*, and *K. oxytoca*	5 (2)
Legese et al. [26]	322	MHT	33	*E. coli, K. pneumoniae*, and Others	4 (12.12)
Beyene et al. [24]	947	MHT	238	*E. coli, K. pneumoniae*, and Others	5 (2)
Mitiku et al. [27]	340	mCIM	59	*E. coli, K. pneumoniae*, and Others	18 (30.5)
Desalegn et al. [30]	873	mCIM	154	*E. coli, K. pneumoniae*, and Others	6 (3.9)
Abdeta et al. [29]	1,337	mCIM	293	*E. coli, K. pneumoniae*, and Others	14 (4.77)
Seman et al. [22]	120	HT and CIM	120	*E. coli, K. pneumoniae, K. oxytoca*, and Others	8 (6.7)
Tekele et al. [16]	312	CIM	312	*E. coli, K. pneumoniae*, and Others	8(2.6)
Seman et al. [23]	2397	CIM	104	*E. coli, K. pneumoniae, K. oxytoca*, and Others	8 (7.7)
Awoke et al. [28]	132	mCIM	132	*K*. *pneumoniae*	28 (21.2)
Aklilu et al. [40]	421	Kirby-Bauer disk diffusion	421	*E. coli, K. pneumoniae*, and Others	6 (1.43)
Zakir et al. [41]	212	mCIM	206	*E. coli, K. pneumoniae*, and Others	5 (2.42)
Eshetie et al. [37]	442	CHROM agar KPC medium	183	*E. coli, K. pneumoniae*, and Others	5 (2.7)
Moges et al. [31]	532	MHT	174	*E. coli, K. pneumoniae*, and Others	23 (13.2)
Alebel et al. [32]	270	mCIM	71	*E. coli, K. pneumoniae*, and Others	12 (16.9)
Moges et al. [35]	833	MHT	133	*E. coli, K. pneumonia*, and Others	8 (6)
Worku et al. [36]	384	mCIM	404	*E. coli, K. pneumoniae*, and Others	4 (1)
Amare et al. [34]	290	mCIM	347	*E. coli, K. pneumoniae*, and Others,	7 (2.4)
Tadesse et al. [33]	384	mCIM	100	*E. coli, E. cloacae, K. pneumoniae*, and Others	6 (6)
Alemayehu et al. [38]	103	mCIM	92	*E. coli, K. pneumoniae* and Others	5 (5.4)
Gashaw et al. [39]	192	Kirby-Bauer disk diffusion	89	*E. coli, K. pneumoniae*, and Others	19 (21.3)

HT, Hodge test; MHT, modified Hodge test; mCIM, modified carbapenem inactivation method; CIM, carbapenem inactivation method. ^#^Others include *Proteus spp*., *K. oxytoca*, *K. ozaenae*, *E. cloacae*, *Citrobacter spp*, *Enterobacter Spp., Salmonella spp*., *Serratia spp*., and *Morganella spp*., No; number of CPE, Prev; prevalence of CPE

### Prevalence of CPE in Ethiopia

**Fig. 2:**
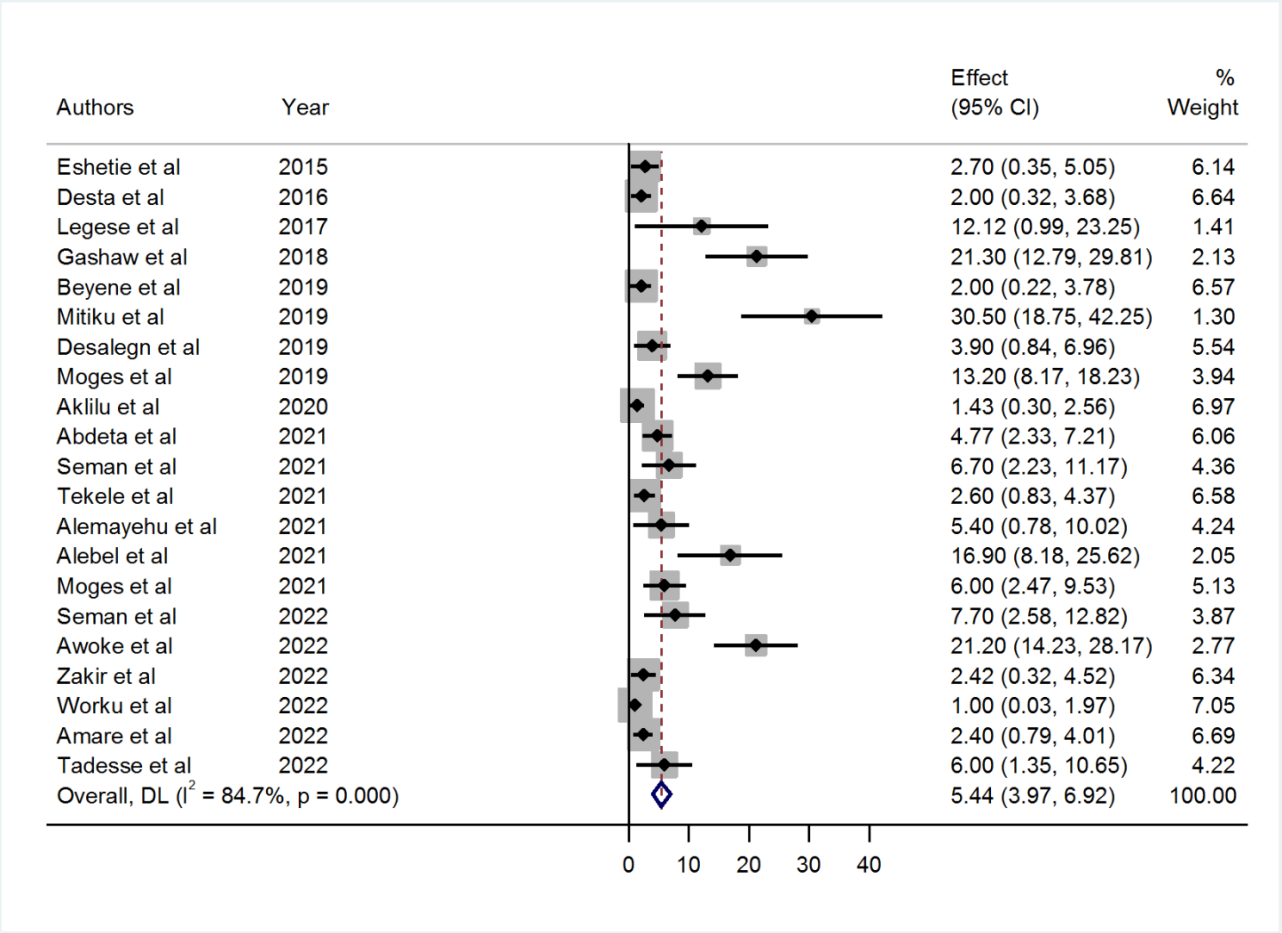
Forest plot showing the pooled prevalence of CPE in Ethiopia from random-effect model analysis

A greater disparity in the prevalence of CPE was revealed in the studies. The prevalence ranges from 1% (95% CI: 0.03, 1.97) reported in Gondar to 30.50% (95% CI: 18.75, 42.25) reported in Addis Ababa. The overall pooled prevalence of CPE in Ethiopia from the random effects model was 5.44% (95% CI: 3.97, 6.92). There was a high level of heterogeneity between studies (I^2^ = 84.7%) and the Q test (Tau-squared = 7.77, p < 0.001) (Fig. 2).

### Subgroup analysis of CPE prevalence in Ethiopia

The subgroup analysis by different regions of Ethiopia indicated that the highest pooled prevalence of 6.45% (95% CI 3.88, 9.02) was observed in Central Ethiopia, followed by 5.27% (95% CI 2.66, 7.88) in the Amhara region. On the other hand, the lowest prevalence of 1.65% (95% CI 0.66, 2.65) was reported in the Southern Nations, Nationalities, and Peoples’ Region (SNNPR). In addition, the subgroup analysis based on city revealed a prevalence of 11.35% (95% CI: 5.15, 17.59) in Bahir Dar, 6.45% (95% CI: 3.88, 9.02) in Addis Ababa, 1.9% (95% CI: 0.73, 3.08) in Gondar, and 1.65% (95% CI: 0.66, 2.65) in Arba Minch. Similarly, subgroup analysis based on the publication year of studies showed that the highest pooled prevalence of 17.44 (95% CI 8.56, 26.32) was reported in 2017–2018 followed by 6.60% (95% CI: 2.66, 10.55) in 2019–2020, and 5.30% (95% CI: 3.39, 7.20) in 2021–2022. The lowest prevalence of 2.24% (95% CI: 0.87, 3.60) was reported in 2015–2016 (Table 3).

**Table 3:** Subgroup analysis of CPE by region, city and year of publication in Ethiopia

Subgroup		No of studies	Pooled prevalence (95% CI)	Heterogeneity test (I^2^)	P-value
Region	Central	10	6.45 (3.88, 9.02)	84.9%	< 0.001
	Amhara	7	5.27 (2.66, 7.88)	85.5%	< 0.001
	SNNPR	2	1.65 (0.66, 2.65)	0.0%	0.416
	Total pooled	19	4.92 (3.49, 6.36)	83.3%	< 0.001
City	Addis Ababa	10	6.45 (3.88, 9.02)	84.9%	< 0.001
	Gondar	4	1.9 (0.73, 3.08)	36.4%	0.194
	Bahir Dar	3	11.35 (5.15, 17.59)	70.4%	0.034
	Arba Minch	2	1.65 (0.66, 2.65)	0.0%	0.416
	Total pooled	19	4.86 (3.42, 6.29)	83.3%	< 0.001
Publication year	2015–2016	2	2.24 (0.87, 3.60)	0.0%	0.635
	2017–2018	2	17.44 (8.56, 26.32)	39.4%	0.199
	2019–2020	5	6.60 (2.66, 10.55)	90.8%	< 0.001
	2021–2022	12	5.30 (3.39, 7.20)	82.9%	< 0.001
	Total pooled	21	5.44 (3.96, 6.92)	83.6%	< 0.001

### Publication bias

**Fig. 3:**
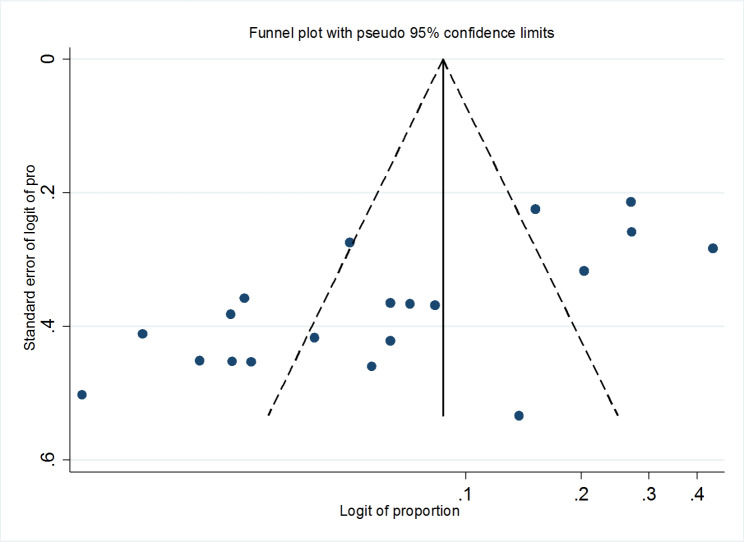
Funnel plot on the prevalence of CPE in Ethiopia

The selected studies were visually evaluated using a funnel plot for possible publication bias. The asymmetry of the funnel plot indicated the presence of publication bias, as more than 66% of the studies fell on the left side of the triangular region (Fig. 3). Furthermore, the result of Egger’s test also revealed a marginally significant publication bias (p < 0.01) (Table 4).

**Table 4:** Egger’s test statistics of the prevalence of CPE in Ethiopia

Std_Eff	Coef.	Std. Err.	T	P> |t|	95% CI
**Slope**	-1.17	0.44	-2.64	0.016	-2.10, -0.24
**Bias**	4.00	0.39	10.23	0.000	3.18, 4.82

### Trim and fill analysis of pooled prevalence of CPE in Ethiopia

A trim and fill analysis was performed due to the presence of publication bias. After adding ten studies, the pooled prevalence of CPE in Ethiopia was 2.31% (95% CI: 0.68–3.94) (Table 5).

**Table 5:** Trim and fill analysis of the prevalence of CPE in Ethiopia

Method	Pooled est.	95% CI		Asymptotic		No. of studies
		Lower	Upper	z-value	p-value	
Fixed	2.556	2.071	3.042	10.323	0.000	21
Random	5.440	3.963	6.916	7.219	0.000	
Test for heterogeneity: Q = 130.257 on 20 degrees of freedom (p = 0.000)						
Moment-based estimate of between studies variance = 7.775						
Trimming estimator: Linear						
Meta-analysis type: Fixed-effects model						
**Iteration**	**Estimate**	**Tn**	**# To trim**		**Diff**	
1	2.556	198	8		231	
2	2.079	213	10		30	
3	1.972	218	10		10	
4	1.972	218	10		0	
Filled						
Meta-analysis						
**Method**	**Pooled est.**	**95% CI**		**Asymptotic**		**No. of studies**
		**Lower**	**Upper**	**z-value**	**p-value**	
Fixed	1.972	1.503	2.442	8.240	0.000	31
Random	2.316	0.689	3.942	2.790	0.005	
Test for heterogeneity: Q = 256.906 on 30 degrees of freedom (p = 0.000)						
Moment-based estimate of between studies variance = 14.681						

### Sensitivity analysis

A sensitivity analysis was carried out using a random effects model to assess the impact of several studies on the combined estimate. The pooled prevalence that was obtained after individual studies were excluded was within the 95% CI of the total pooled estimate. This demonstrates that no single study had an impact on the total pooled effect magnitude (Table 6).

**Table 6:** Sensitivity analysis of the prevalence of CPE in Ethiopia

Study omitted	Estimate	95% CI
Seman et al. [22]	5.37	3.86, 6.88
Seman et al. [23]	5.33	3.83, 6.82
Legese et al. [26]	5.32	3.84, 6.80
Tekele et al. [16]	5.75	4.16, 7.34
Awoke et al. [28]	4.74	3.38, 6.09
Zakir et al. [41]	5.73	4.16, 7.30
Aklilu et al. [40]	5.94	4.30, 7.58
Tadesse et al. [33]	5.41	3.90, 6.93
Abdeta et al. [29]	5.51	3.97, 7.05
Amare et al. [34]	5.79	4.19, 7.39
Moges et al. [35]	5.41	3.89, 6.92
Worku et al. [36]	5.94	4.32, 7.57
Eshetie et al. [37]	5.69	4.12, 7.25
Alemayehu et al. [38]	5.45	3.93, 6.96
Gashaw et al. [39]	4.93	3.53, 6.33
Beyene et al. [24]	5.79	4.20, 7.38
Desta et al. [25]	5.80	4.21, 7.40
Mitiku et al. [27]	4.92	3.54, 6.30
Desalegn et al. [30]	5.56	4.02, 7.10
Moges et al. [31]	4.96	3.54, 6.39
Alebel et al. [32]	5.11	3.67, 6.55
Combined	5.43	3.96, 6.91

## Discussion

The current systematic review and meta-analysis was carried out to determine the pooled prevalence of CPE in Ethiopia. Antibiotic resistance among Enterobacteriaceae has been widely reported and has grown to represent a serious threat to the delivery of healthcare [42]. Due to their high levels of antibiotic resistance, carbapenemase-producing Enterobacteriaceae (CPE) are challenging to treat since they are able to break down all beta-lactam medicines, including carbapenems, and render them ineffective [43]. Their high prevalence may also result in higher mortality, longer hospital stays, and increased consumption of healthcare services [44, 45]. Estimating the pooled prevalence of CPE is therefore a critical step to offering information on the temporal and geographic incidence of carbapenem resistance, as well as the extent of the problem, in order to develop a national public health response to these emerging pathogens.

In this systematic review and meta-analysis, the pooled prevalence of CPE in Ethiopia was 5.44% (95% CI: 3.97, 6.92). However, it was varied to 2.31% (95% CI: 0.68, 3.94) by adding ten studies to the trim and fill analysis. The observed high carbapenem resistance rate could be due to prior antimicrobial exposure, a history of hospitalization, the length of hospital stays, the presence of invasive devices, advanced age, and severe underlying diseases [46]. It could also potentially be attributable to drugs being prescribed without awareness of their susceptibility pattern or the introduction and spread of carbapenem-resistant bacterial strains from other places with high resistance rates. Repetitive, improper, and inaccurate use of antimicrobial drugs in empirical treatment, as well as inadequate infection control techniques, may also increase the prevalence of carbapenem resistant Enterobacteriaceae in the population.

The pooled estimate is comparable with the reports from Kuwait (4.9%) [47], Lebanon (5.19%) [48], Malaysia (5.74%) [49], Senegal (5.1%) [50], and the United Arab Emirates (4.6%) [51]. On the other hand, the finding is higher when compared to the findings of previous reports from eighteen European nations (2%) [52], South Korea (1.6%) [53], Belgium (3.5%) [54], Lebanon (3%) [55], and Afghanistan (3.4%) [56]. Nevertheless, the pooled prevalence report was lower than reports from Kuwait (8%) [57], Saudi Arabia (23.9%) [58], and Egypt (54.1%) [59]. This difference could be because of the use of different antibiotic susceptibility testing (AST) methods, target population, sample type, type and number of bacteria isolates, the definition used to classify presence of carbapenemase-producing isolates, antibiotic use policy variations, and geographical area. Additionally, the discrepancy could be attributed to differences in local antibiotic prescribing habits and infection control programs in various health care facilities [60].

The high degree of heterogeneity in the overall prevalence found in our analysis could be caused by a number of factors. As a result, we took into account post-hoc subgroup analysis by many factors, including region, city, and publication year of the study. Variations in the prevalence of CPE were observed in different regions of Ethiopia, with the highest in Central Ethiopia (6.45%) and the lowest in the SNNPR region (1.65%). Moreover, the sub-group analysis by city found the highest CPE prevalence in Bahir Dar (11.35%) and the lowest in Arba Minch (1.65%). Finally, the sub-group analysis by publication year of the study indicated that the highest prevalence of CPE was found in 2017–2018 (17.44%) and the lowest in 2015–2016 (2.24%). This discrepancy might be attributable to the study period, environmental factors, target population, type of sample, and type and number of bacteria isolates. Some factors were also mentioned as one of the reasons for the discrepancy in the prevalence of carbapenem resistant Enterobacteriaceae.

This review has certain strengths and limitations. It involved more than one reviewer. In addition, we employed a comprehensive search technique and attempted to investigate grey literature. Moreover, during this review, we have also strictly followed the PRISMA guidelines. However, our meta-analysis has limitations, such as the presence of significant heterogeneity even after subgroup analysis for some variables. Also, only studies published in English were included, which may expose the study to language bias. As a result, the meta-analysis revealed significant heterogeneity, with some CIs overlapping in the subgroup analysis. So, some estimations could be impacted by group interaction. On the other hand, it was unable to assess factors associated with the pooled prevalence of CPE. Furthermore, all of the studies included in this systematic review and meta-analysis were cross-sectional studies, and the outcome variability may be influenced by other confounding variables. These limitations may have an impact on the findings reported in this review regarding the overall prevalence of CPE in Ethiopia.

## Conclusion

This systematic review and meta-analysis showed a high prevalence of carbapenemase-producing Enterobacteriaceae in Ethiopia. As a result, steps should be taken to reduce the spread of CPE. Resistance to third-generation cephalosporins is also a major issue. However, it is necessary to improve the infection prevention strategy and conduct additional national surveillance on the profile of carbapenemase production and their determining genes among Enterobacteriaceae clinical isolates.

## Electronic supplementary material

Below is the link to the electronic supplementary material.

Supplementary Material 1

## Data Availability

All the datasets used and/or analyzed during the current study are available in the manuscript.

## Abbreviations

CLSI: Clinical Laboratory Standards Institute
CPE: Carbapenemase-Producing Enterobacteriaceae
CRE: Carbapenem Resistant Enterobacteriaceae
SNNPR: Southern Nations, Nationalities, and Peoples’ Region
